# Supplementary material for: The HER2MtGx Metagene Score as a Reliable Tool to Select HER2 Breast Cancer Patients for Neoadjuvant Targeted Therapy
Source: Int J Mol Sci. 2025 Dec 6;26(24):11809. doi: 10.3390/ijms262411809 (PMC12732813; doi:10.3390/ijms262411809)
Supplement: Supplementary file 1 [file ijms-26-11809-s001.zip › ijms-3970498-supplementary.pdf]

SUPPLEMENTAL MATERIAL

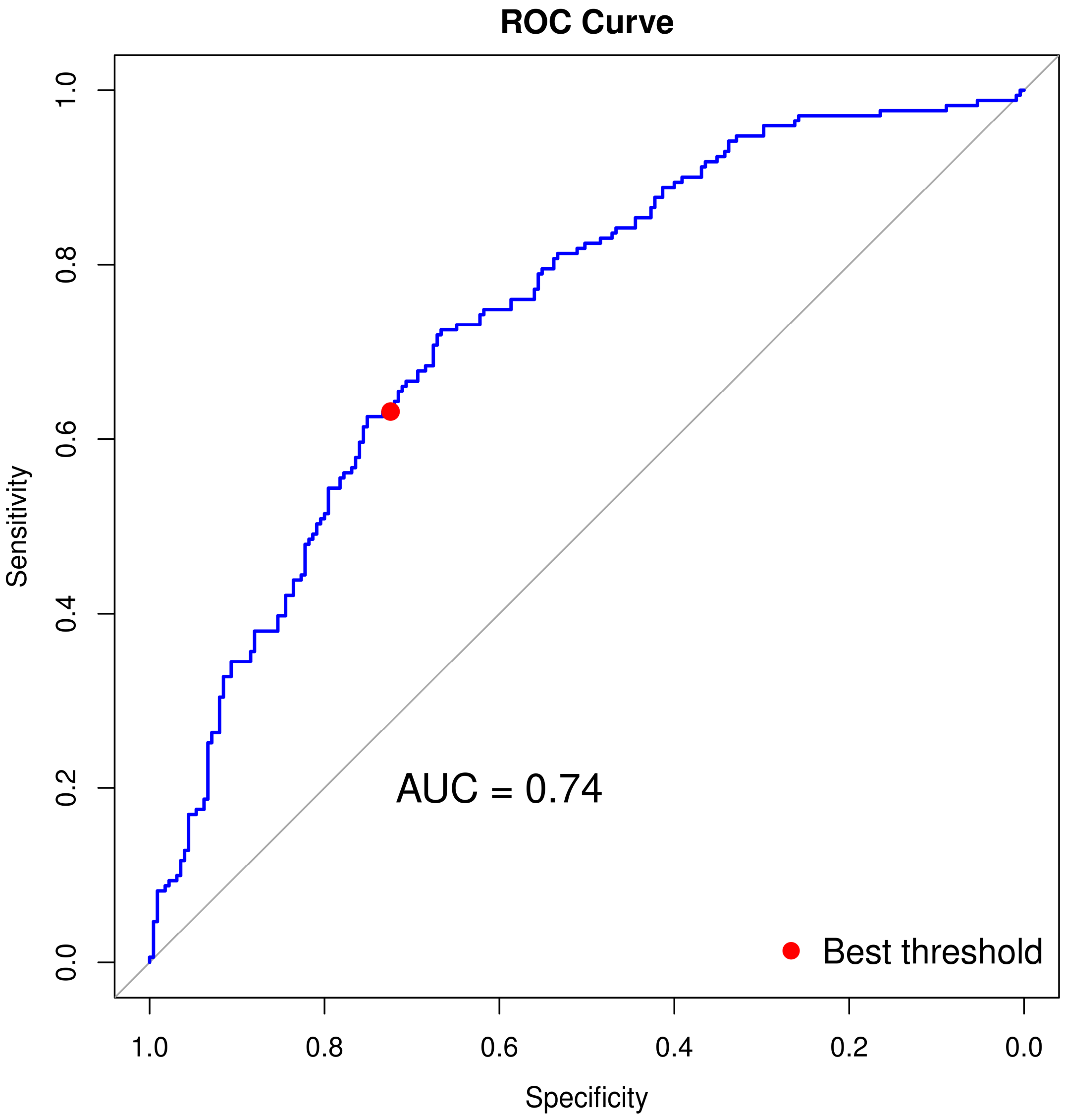

Figure S1. ROC plot displaying the sensitivity and specificity of the HER2MtGx model as a pCR prediction in all patients subjected to NACT + target therapy for the I-SPY, NOAH and CHER-LOB cohorts.

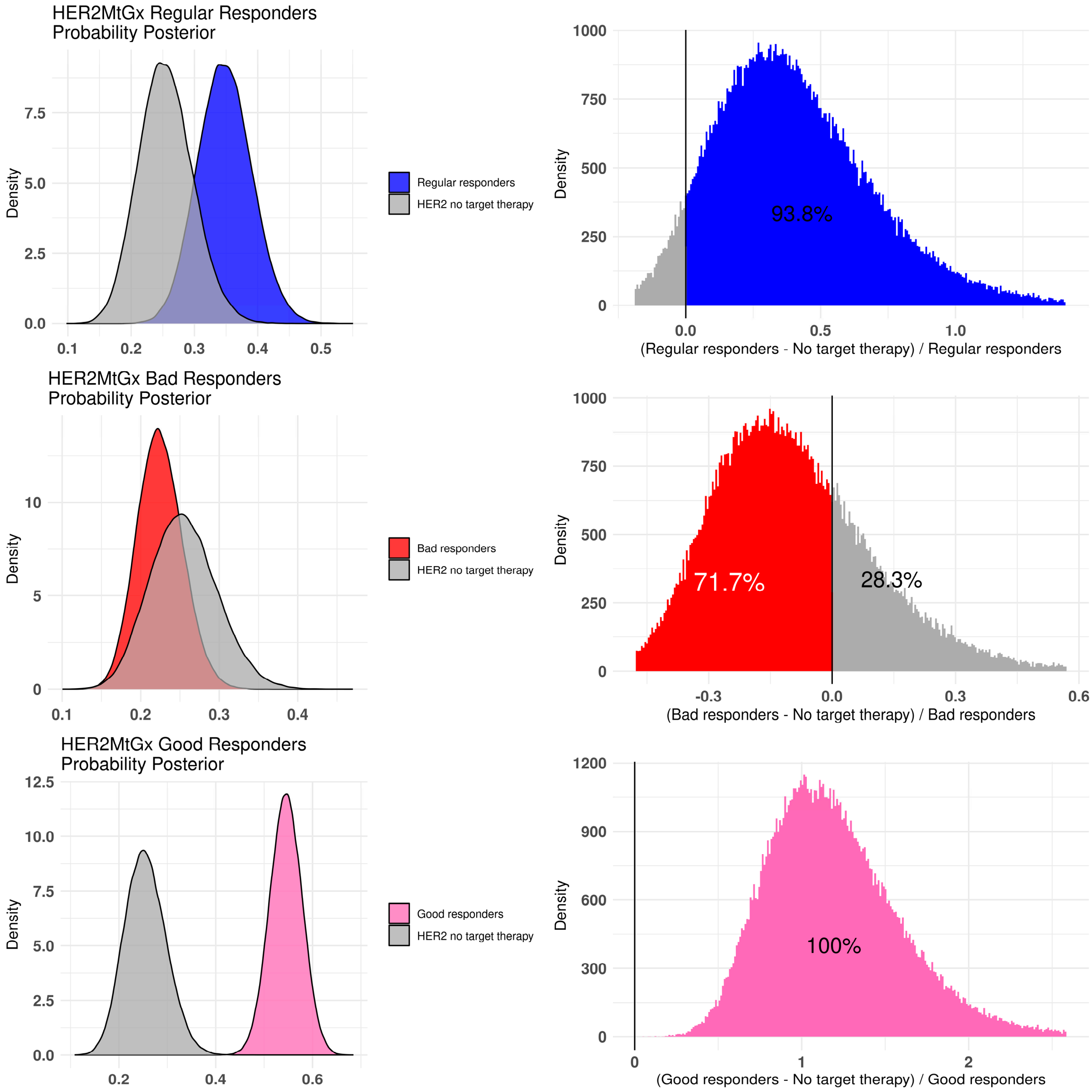
HER2MtGx Regular Responders
Probability Posterior
Density
Regular responders
HER2 no target therapy
93.8%
(Regular responders - No target therapy) / Regular responders
HER2MtGx Bad Responders
Probability Posterior
Bad responders
HER2 no target therapy
71.7%
28.3%
(Bad responders - No target therapy) / Bad responders
HER2MtGx Good Responders
Probability Posterior
Good responders
HER2 no target therapy
100%
(Good responders - No target therapy) / Good responders

Figure S2. Bayesian inference representation according to the response classification in all cHER2+ samples from all neoadjuvant cohorts (I-SPY, NOAH and CHER-LOB). On the left, the posterior probability distributions to achieve pCR are represented for the response group compared to cHER2+ treated with NACT without target therapy. On the right the density plot expresses the probability of achieving pCR with NACT plus target therapy compared to the NACT without target therapy. Note that adding target therapy has a 100% chance to substantially increase the pCR rate in patients with samples classified as good responders. In the bad responders, the probability of achieving pCR rate is lower than the baseline.

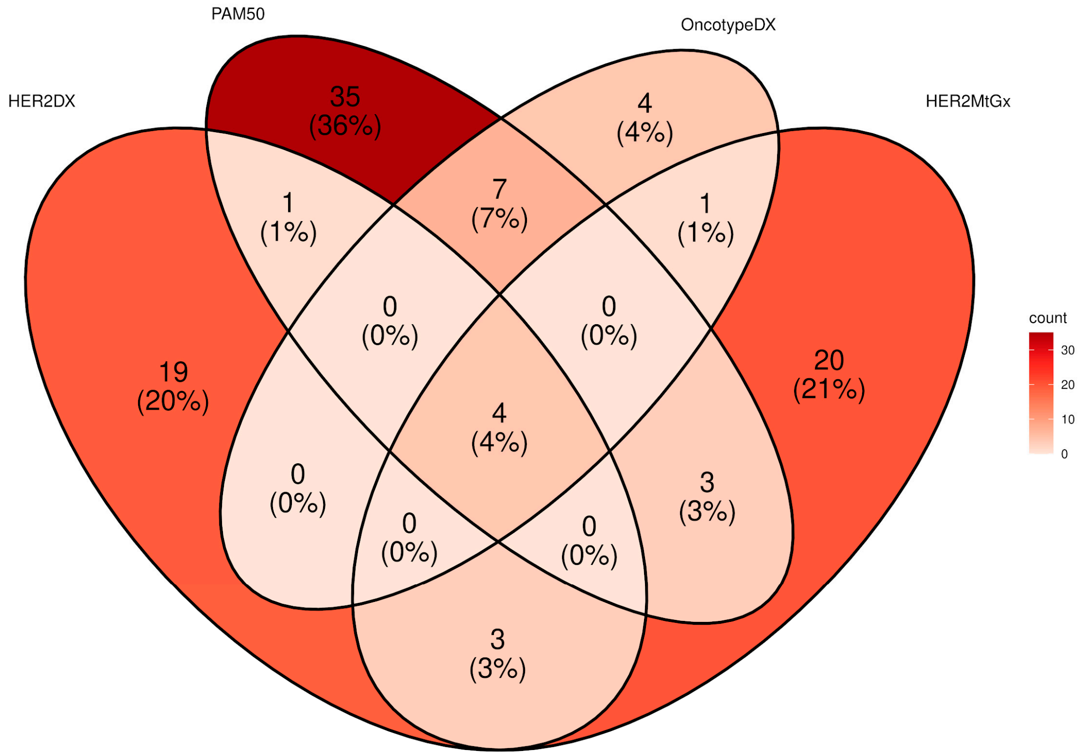

Figure S3. Venn diagram representing the number of common genes among HER2MtGx, HER2DX, PAM50 and OncotypeDx assays.

Table S1. *Post hoc* statistics (Dunn's test) comparing the amplicon copy number (log_2 ratio average) among all the 5 breast cancer clusters. The cluster 5 represents the HER2 enriched samples.

Pairs	Z	P	P-adj
1 - 2	-1.3	0.1	0.2
1 - 3	1.7	0.07	0.1
**2 - 3**	**2.7**	**0.006**	**0.01**
1 - 4	-0.2	0.8	0.8
2 - 4	0.8	0.4	0.4
3 - 4	-1.6	0.09	0.1
**1 - 5**	**-6.5**	**<0.0001**	**<0.0001**
**2 - 5**	**-4.5**	**<0.0001**	**<0.0001**
**3 - 5**	**-6.6**	**<0.0001**	**<0.0001**
**4 - 5**	**-4.5**	**<0.0001**	**<0.0001**

Table S2. The scores were divided into quantiles and the pCR rate was analyzed by quantiles according to each score (Her2, Basal and Luminal). Note the higher Her2 score and the lower Luminal score the higher the pCR rate. The Basal score is not associated with pCR rate.

Characteristic	**Q1** N = 307[1]	**Q2** N = 308[1]	**Q3** N = 308[1]	**Q4** N = 308[1]	p-value[2]
**pCR: Her2 score**					**<0.001**
0	241 (79%)	221 (72%)	214 (69%)	156 (51%)	
1	66 (21%)	87 (28%)	94 (31%)	152 (49%)	
**pCR: Basal score**					0.2
0	198 (64%)	212 (69%)	222 (72%)	201 (65%)	
1	109 (36%)	96 (31%)	86 (28%)	107 (35%)	
**pCR: Luminal score**					**<0.001**
0	173 (56%)	184 (60%)	206 (67%)	269 (87%)	
1	134 (44%)	124 (40%)	102 (33%)	39 (13%)	

[1]n (%)

[2]Pearson's Chi-squared test

pCR = pathological complete response; Q1 to Q4 = scores quantiles

Table S3. PAM50 and HER2MtGx models accuracies and predictive values comparison using the interval for binomial proportions.

model	metric	estimate	lower ci	upper ci	p
PAM50	Sensitivity	62.4	54.2	70	ns
HER2MtGx	Sensitivity	76.6	69	82.9	0.001
PAM50	Specificity	72.1	65.3	78.2	ns
HER2MtGx	Specificity	66.1	59	72.6	ns
PAM50	PPV	63.3	55.1	70.9	ns
HER2MtGx	PPV	63.5	56.1	70.4	ns
PAM50	NPV	71.4	64.5	77.4	ns
HER2MtGx	NPV	78.6	71.5	84.4	0.001
PAM50	PLR	2.2	1.7	2.9	ns
HER2MtGx	PLR	2.3	1.8	2.8	ns
PAM50	NLR	0.5	0.4	0.7	0.001
HER2MtGx	NLR	0.4	0.3	0.5	ns

Ns= not significant; PPV= Positive Predictive Value; NPV= Negative Predictive Value; PLR= Positive Likelihood Ratio; NLR= Negative Likelihood Ratio
